# Long non-coding RNA MALAT1 acts as a competing endogenous RNA to promote malignant melanoma growth and metastasis by sponging miR-22

**DOI:** 10.18632/oncotarget.11564

**Published:** 2016-08-24

**Authors:** Wenkang Luan, Lubo Li, Yan Shi, Xuefeng Bu, Yun Xia, Jinlong Wang, Henry Siaw Djangmah, Xiaohui Liu, Yongping You, Bin Xu

**Affiliations:** ^1^ Department of Plastic Surgery, Affiliated People's Hospital of Jiangsu University, Zhenjiang, Jiangsu, China; ^2^ Department of Neurosurgery, Affiliated People's Hospital of Jiangsu University, Zhenjiang, Jiangsu, China; ^3^ Department of General Surgery, Affiliated People's Hospital of Jiangsu University, Zhenjiang, Jiangsu, China; ^4^ Department of Neurosurgery, Nanjing First Hospital, Nanjing Medical University, Nanjing, China; ^5^ Department of Neurosurgery, The First Affiliated Hospital of Nanjing Medical University, Nanjing, China

**Keywords:** MALAT1, miR-22, MMP14 and Snail, ceRNA, melanoma

## Abstract

Long non-coding RNAs (lncRNAs) are involved in tumorigenesis. Metastasis-associated lung adenocarcinoma transcript 1 (MALAT1), an lncRNAs, is associated with the growth and metastasis of many human tumors, but its biological roles in malignant melanoma remain unclear. In this study, the aberrant up-regulation of MALAT1 was detected in melanoma. We determined that MALAT1 promotes melanoma cells proliferation, invasion and migration by sponging miR-22. MiR-22 was decreased and acted as a tumor suppressor in melanoma, and MMP14 and Snail were the functional targets of miR-22. Furthermore, MALAT1 could modulate MMP14 and Snail by operating as a competing endogenous RNA (ceRNA) for miR-22. The effects of MALAT1 in malignant melanoma is verified using a xenograft model. This finding elucidates a new mechanism for MALAT1 in melanoma development and provides a potential target for melanoma therapeutic intervention.

## INTRODUCTION

Malignant melanoma, the most aggressive of all skin cancers, is the primary cause of skin cancer deaths, with an increasing incidence worldwide [1–3]. The generation and development of melanoma involves complex changes in networks of genes, signaling pathways, and gene regulation that control tumor cell proliferation, apoptosis, invasion, and migration [4–6]. Thus, the underlying molecular mechanisms of melanoma need to be further studied.

Non-coding RNAs (ncRNAs) are new regulatory molecule, the differential expression of which contributes to tumorigenesis, including melanoma [7, 8]. Extensive reports demonstrated that microRNAs (miRNAs), one of the best-studied ncRNAs, act as oncomiRNAs or tumor suppressors in melanoma [9, 10]. In recent years, the role of long non-coding RNAs (LncRNAs) in tumor development has received increasing attention. The aberrant expression of lncRNAs is associated with melanoma genesis and participates in different biological processes in melanoma [11, 12]. Nevertheless, only a few lncRNAs functions and mechanisms have been identified in melanoma.

Metastasis-associated lung adenocarcinoma transcript 1 (MALAT1), also known as nuclear-enriched transcript 2 (NEAT2), was first identified in the study of the metastasis of non-small cell lung cancer [13]. Accumulating evidence has shown that MALAT1 contributes to proliferation and metastasis in many human tumors [14, 15]. Melanoma also over-expresses MALAT1 [16]. Increased expression of MALAT1 may promote melanoma metastasis [16]. However, the function and molecular mechanism of MALAT1 in melanoma remains unclear. In this study, we showed that the expression of MALAT1 was increased in melanoma patients. We also demonstrated that MALAT1 promoted the proliferation, invasion and migration of melanoma cells by acting as a competing endogenous RNA (ceRNA) for miR-22. MiR-22 suppressed tumor invasion and metastasis by targeting member matrix metalloproteinase 14 (MMP14) and Snail [17]. Subsequently, we demonstrated that MMP14 and Snail were the downstream target of MALAT1 ceRNA function and were important for MALAT1 to regulate melanoma cell phenotypes, suggesting that MALAT1 affected melanoma progression in a miR-22 site-dependent manner. Thus, these results suggest that MALAT1 regulates melanoma genesis as a ceRNA and may serve as the potential target for melanoma therapy.

## RESULTS

### MALAT1 is significantly over-expressed in melanoma and promotes the proliferation, invasion and migration of melanoma cells

**Figure 1:**
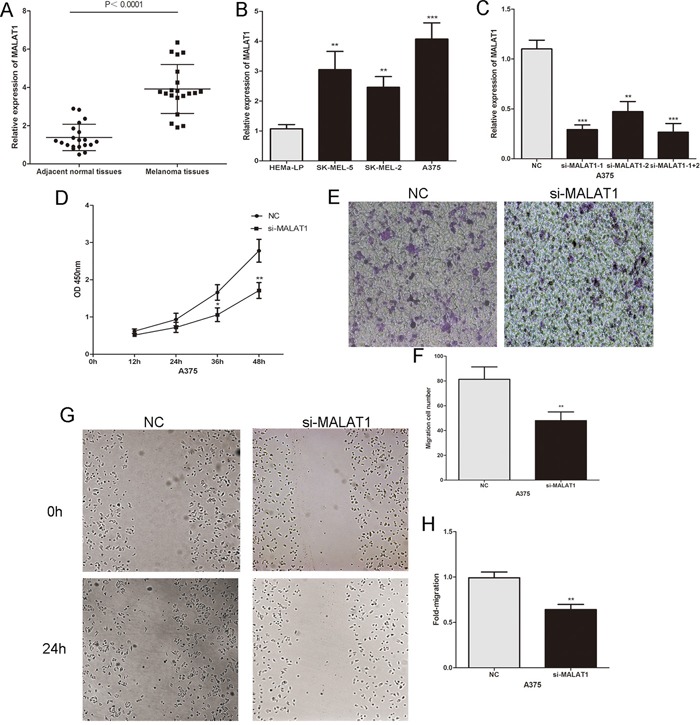
The expression and the biological functions of MALAT1 in melanoma **A.** MALAT1 levels were analysed in primary malignant melanoma tissues and adjacent normal tissues. **B.** MALAT1 expression profile in human epidermal melanocytes (HEMa-LP) and three human melanoma cell lines (A375, SK-MEL-5 and SK-MEL-2). **C.** Transfection efficiency of si-MALAT1 was determined by PCR. **D.** The proliferative ability of melanoma cell lines A375 was measured by CCK8 assay after the cells were transfected with si-MALAT1 or NC. **E.** and **F.** The effect of si-MALAT1 on the invasive capacity of melanoma cells was assessed by transwell assay. **G.** and **H.** The effect of si-MALAT1 on the migratory ability of melanoma cells was assessed by the scratch wound assay. *P < 0.05, **P < 0.01, ***P<0.001. The results are representative of at least three independent experiments.

We initially investigated MALAT1 expression in twenty pairs of primary malignant melanoma tissues and adjacent normal tissues by real-time PCR. As shown in Figure 1A, MALAT1 was markedly increased in melanoma tissues compared to adjacent normal tissues. We also detected the expression of MALAT1 in human epidermal melanocytes (HEMa-LP) and three human melanoma cell lines (A375, SK-MEL-5 and SK-MEL-2), and found that MALAT1 was significantly higher in melanoma cells than in human epidermal melanocytes, especially in A375 cells (Figure 1B). To further explore the role of MALAT1 in melanoma cells, MALAT1 was down-regulated by si-MALAT1 in A375 cells (Figure 1C). The CCK8 assay revealed that decreased MALAT1 significantly inhibited the proliferative ability of melanoma cells (Figure 1D). Scratch wound assays and transwell assays showed that si-MALAT1 transfected cells exhibited a decrease in invasive and migratory capacity compared to the control group (Figure 1E–1H). These findings indicated that MALAT1 promotes tumor cell proliferation, migration and invasion in melanoma.

### MALAT1 is physically associated with miR-22 in melanoma cells

**Figure 2:**
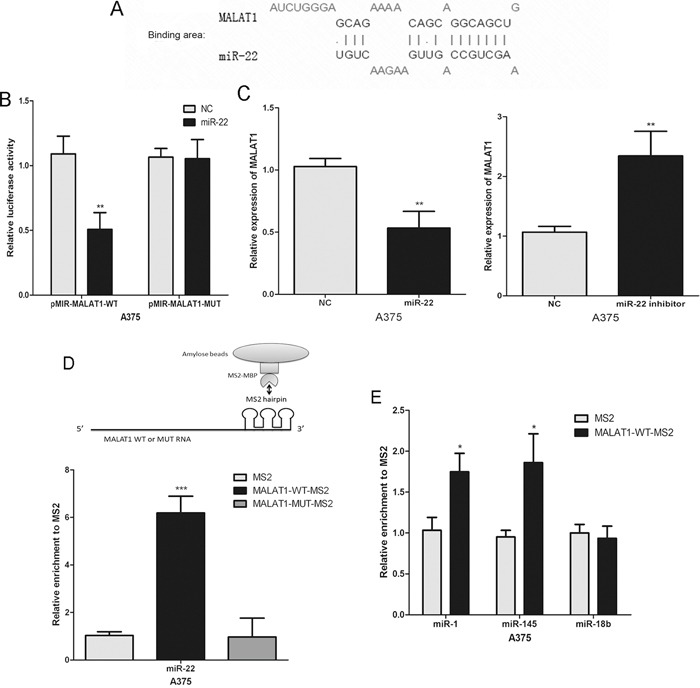
The interaction of MALAT1 with miR-22 **A.** The putative binding sites of miR-22 on the MALAT1 transcript, as predicted by LncBase Predicted v.2. **B.** Over-expression of miR-22 led to a marked decrease in luciferase activity of pMIR-MALAT1-WT, without any change in luciferase activity of pMIR-MALAT1-MUT in A375 cells. **C.** The expression levels of MALAT1 in A375 cells transfected with the miR-22 mimic or inhibitor. **D.** and **E.** MS2-RIP followed by miRNA RT-PCR to detect endogenous miR-22, miR-1, miR-18b or miR-145 associated with the MS2-tagged MALAT1 transcript. *P < 0.05, **P < 0.01, ***P<0.001. The results are representative of at least three independent experiments.

Several studies indicated that lncRNA may operate as a ceRNA in carcinogenesis. The hypothesis of ceRNA function assumes that some specific lncRNAs can function as miRNA sponges to control miRNAs available for binding with their targets, functionally liberating mRNA transcripts targeted by certain miRNAs [18, 19]. A recent study showed that MALAT1 promotes breast cancer (miR-1) [20], liver fibrosis [21] and cervical cancer (miR-145) [22] progression by acting as a ceRNA. Thus, we considered whether MALAT1 has the same effect in melanomagenesis. The potential miR-22 binding sites in MALAT1 transcripts were predicted using LncBase Predicted v.2 (http://carolina.imis.athena-innovation.gr/index.php?r=site%2Ftools) (Figure 2A). We subsequently constructed luciferase reporter plasmids containing the wild-type MALAT1 (pMIR-MALAT1-WT) and mutant MALAT1 with mutations of predicted miR-22 binding sites (pMIR-MALAT1-MUT). Dual luciferase reporter assays showed that miR-22 over-expression led to a marked decrease in luciferase activity in pMIR-MALAT1-WT, without changing the luciferase activity of pMIR-MALAT1-MUT in A375 cells (Figure 2B). Meanwhile, real-time PCR showed that endogenous MALAT1 was reduced in miR-22 mimic-transfected cells, but miR-22 inhibitor increased MALAT1 levels (Figure 2C). These data indicate that MALAT1 is negatively regulated by miR-22 in melanoma cells.

Subsequently, we performed an RNA pull-down analysis to detect endogenous miRNAs associated with the MALAT1 to further validate the direct interaction between miR-22 and MALAT1. The precipitated miRNAs were analysed by real-time PCR. We found that the MS2-tagged wild-type MALAT1 (MALAT1-WT-MS2) was significantly enriched for miR-22 compared to the empty vector and MALAT1 with a mutation in the miR-22 binding site (MALAT1-MUT-MS2) in A375 cells (Figure 2D). However, the biding between MALAT1 and miR-1 or miR-145 (positive control) were significantly less than miR-22 (Figure 2E). We predicted there are no miR-18b (a tumour suppressor in melanoma) binding site in MALAT1 transcripts. MiR-18b was used as a negative control in the pull down assays, and with no statistically significant (Figure 2E). These findings suggested that miR-22 was MALAT1 primary targeting endogenous miRNAs in melanoma cells.

### MiR-22 is down-regulated in melanoma tissues and represses melanoma cells proliferation, invasion and migration

**Figure 3:**
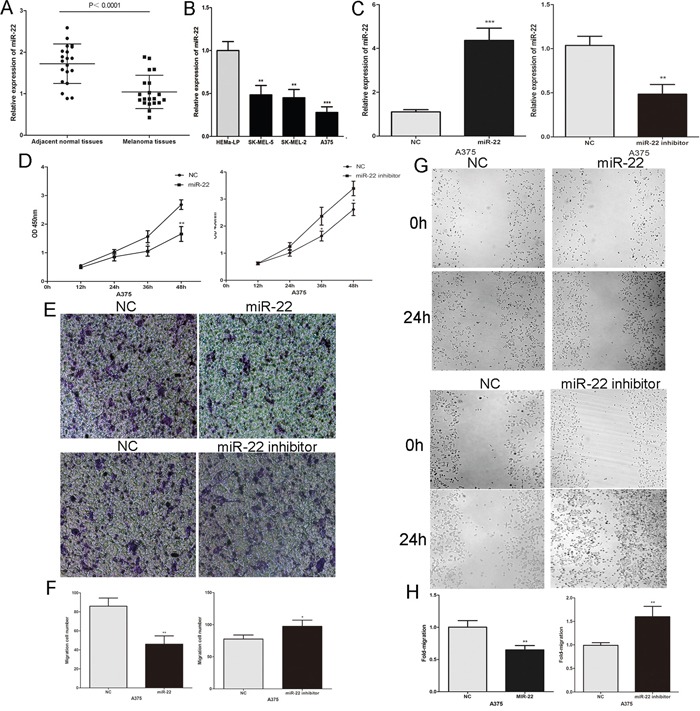
The expression and the biological functions of miR-22 in melanoma **A.** The level of miR-22 was analysed in primary malignant melanoma tissues and adjacent normal tissues. **B.** MiR-22 expression profile in human epidermal melanocytes (HEMa-LP) and three human melanoma cell lines (A375, SK-MEL-5 and SK-MEL-2). **C.** Transfection efficiency of the miR-22 mimic or inhibitor were determined by PCR. **D.** The proliferative ability of melanoma cell measured by the CCK8 assay. **E.** and **F.** The effect of miR-22 mimic or inhibitor on the invasive capacity of melanoma cells was assessed by the transwell assay. **G.** and **H.** The effect of miR-22 mimic or inhibitor on the migratory ability of melanoma cells was assessed by the scratch wound assay. *P < 0.05, **P < 0.01, ***P<0.001. The results are representative of at least three independent experiments.

The above findings indicate that it is essential to investigate the role of miR-22 in melanoma. We first detected miR-22 expression in 20 pairs of malignant melanoma tissues and adjacent normal tissues by real-time PCR. The miR-22 level was significantly reduced in melanoma tissues compared to adjacent normal tissues (Figure 3A). Then we detected the expression of miR-22 in human epidermal melanocytes and human melanoma cell lines, and found that miR-22 was significantly lower in melanoma cells than in human epidermal melanocytes (Figure 3B). The miR-22 mimic or inhibitor was transfected into A375 cells to further study the function of miR-22 in melanoma cells (Figure 3C). Over-expression of miR-22 suppressed the proliferation of A375 melanoma cells, the opposite result was obtained after transfection with miR-22 inhibitor (Figure 3D). Furthermore, the invasive and migratory abilities of A375 melanoma cells were inhibited by the miR-22 mimic, the effect of miR-22 inhibitor is opposite (Figure 3E–3H). Taken together, these results suggest that miR-22 acts as a tumor suppressor in melanoma.

### MMP14 and Snail are the direct functional targets of miR-22 in melanoma cells

**Figure 4:**
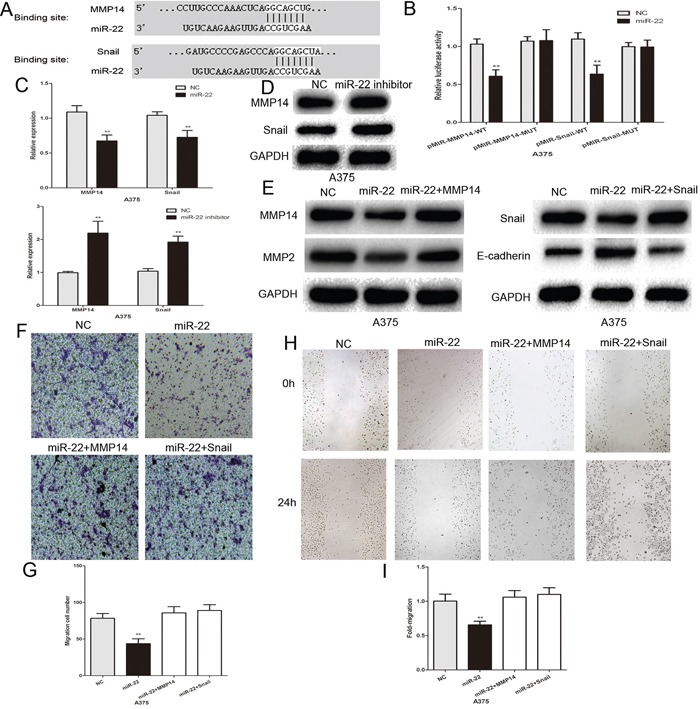
MMP14 and Snail are the functional targets of miR-22 that affect invasive and migratory abilities of melanoma cells **A.** The putative binding sites of miR-22 within the 3′-UTR of MMP14 and Snail, as predicted by TargetScan. **B.** Over-expression of miR-22 led to a marked decrease in luciferase activity of the WT plasmid, without significant change in the MUT plasmid in A375 cells. **C.** PCR to assess the mRNA level of MMP14 and Snail in A375 cells following transfection with miR-22 mimic or inhibitor. **D.** Western blots identified MMP14 and Snail protein expression changes following transfection with miR-22 inhibitor or NC. GAPDH is shown as a loading control. **E.** Western blots identified MMP14, Snail, E-cadherin and MMP2 protein expression changes following transfection with miR-22 alone or in combination with MMP14 and Snail. GAPDH is shown as a loading control. **F.** and **G.** MMP14 and Snail plasmids reversed the effect of miR-22 on the invasive ability of melanoma cells. **H.** and **I.** MMP14 and Snail plasmids reversed the effect of miR-22 on the migratory capacity of melanoma cells. *P < 0.05, **P < 0.01, ***P<0.001. The results are representative of at least three independent experiments.

To study the mechanism of action of miR-22 in melanoma cells, we found that the binding sites of miR-22 matched the 3′-UTR of MMP14 and Snail by using bioinformatic analysis (TargetScan, http://www.targetscan.org/) (Figure 4A). This suggesed that MMP14 and Snail are the potential targets of miR-22. Subsequently, we generated the luciferase reporter plasmids containing the wild-type (pMIR-MMP14-WT and pMIR-Snail-WT) and mutant (pMIR-Snail-MUT and pMIR-Snail-MUT) binding sites of the MMP14 and Snail 3′-UTR. Dual luciferase reporter assays showed that over-expression of miR-22 led to a marked decrease in luciferase activity of the WT plasmid, without significant change in the MUT plasmid in A375 cells (Figure 4B). Real-time PCR and western blotting showed that the expression of endogenous MMP14 and Snail was lower in miR-22 mimic transfected cells, and was increased in the miR-22 inhibitor group (Figure 4C–4E). MMP14 has been shown to play a critical role in cancer invasion by inducing ECM degradation and increasing the secretion of other MMPs such as pro-MMP2 [23, 24]. Snail promotes tumor invasion and metastasis, strongly repressing E-cadherin expression and inducing EMT [25]. We detected the MMP2 and E-cadherin expression using western blotting. MiR-22 up-regulation led to a decrease in MMP2 protein expression and an increase in E-cadherin protein expression, and the change in these protein levels was reversed by MMP14 and Snail (Figure 4E). The effect of miR-22 on the invasive and migratory abilities of A375 melanoma cells was also largely abrogated by the MMP14 and Snail plasmids (Figure 4F–4I). These results indicate that MMP14 and Snail are the functional targets of miR-22 in melanoma cells.

### MALAT1 promotes melanoma cell growth and metastasis by acting as a ceRNA

**Figure 5:**
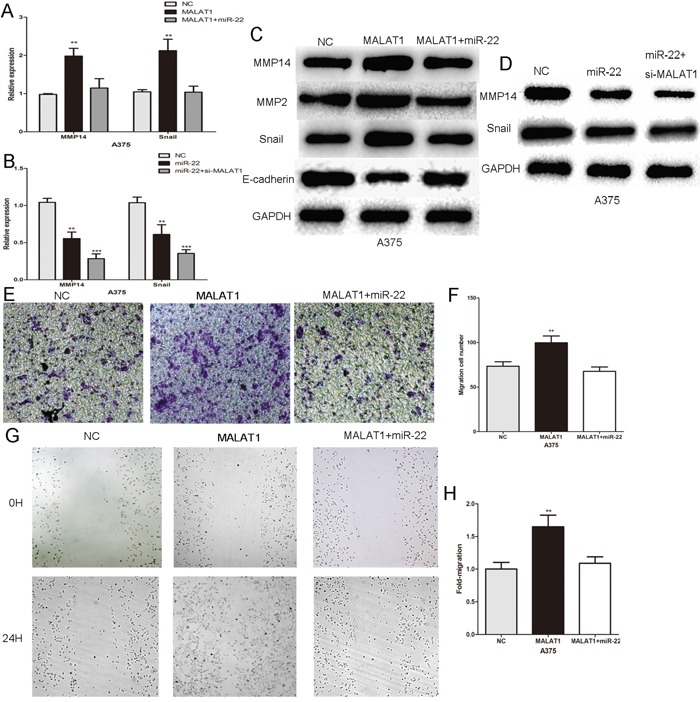
MALAT1 promotes melanoma cell growth and metastasis by acting as a ceRNA **A.** The expression of MMP14 and Snail mRNA in A375 cells transfected with MALAT1 or MALAT1 combination with the miR-22 mimic. **B.** The expression of MMP14 and Snail mRNA transfected with miR-22 or miR-22 combination with si-MALAT1. **C.** Western blots identified MMP14, Snail, E-cadherin and MMP2 protein expression changes following transfection with MALAT1 or MALAT1 in combination with the miR-22 mimic. **D.** Western blots identified MMP14 and Snail protein expression changes following transfection with miR-22 or miR-22 in combination with si-MALAT1. **E.** and **F.** The effect of MALAT1 on the invasion of melanoma cells was largely abrogated by the miR-22 mimic. **G.** and **H.** The miR-22 mimic reversed the effect of MALAT1 on the migratory capacity of melanoma cells. *P < 0.05, **P < 0.01, ***P<0.001. The results are representative of at least three independent experiments.

Using bioinformatic analysis, we determined that MALAT1, a ceRNA, shares regulatory miR-22 with its target MMP14 and Snail (Figure 2A and Figure 4A). We further investigated whether MMP14 and Snail expression could be influenced by MALAT1 in melanoma cells. In A375 cells, MALAT1 over-expression increased the mRNA and protein levels of MMP14 and Snail, with downstream effects on MMP2 and E-cadherin protein expression (Figure 5A and 5C). The influence of MALAT1 on MMP14, Snail, MMP2 and E-cadherin expression was reversed by the miR-22 mimic (Figure 5A and 5C). Meanwhile, Real-time PCR and western blotting showed that the inhibitory effect of miR22 on the MMP14 or Snail expression were more significantly in the absence of MALAT1 (Figure 5B and 5D). Moreover, the effect of MALAT1 on the migration and invasion of melanoma cells was also largely abrogated by an miR-22 mimic (Figure 5E–5H). In summary, the results indicated an important role for MALAT1 in modulating MMP14 and Snail by competitively binding miR-22. MALAT1 promoted melanoma cell growth and metastasis by operating as a ceRNA for the miR-22.

### Inhibition of endogenous MALAT1 expression inhibits melanoma growth *in vivo*

**Figure 6:**
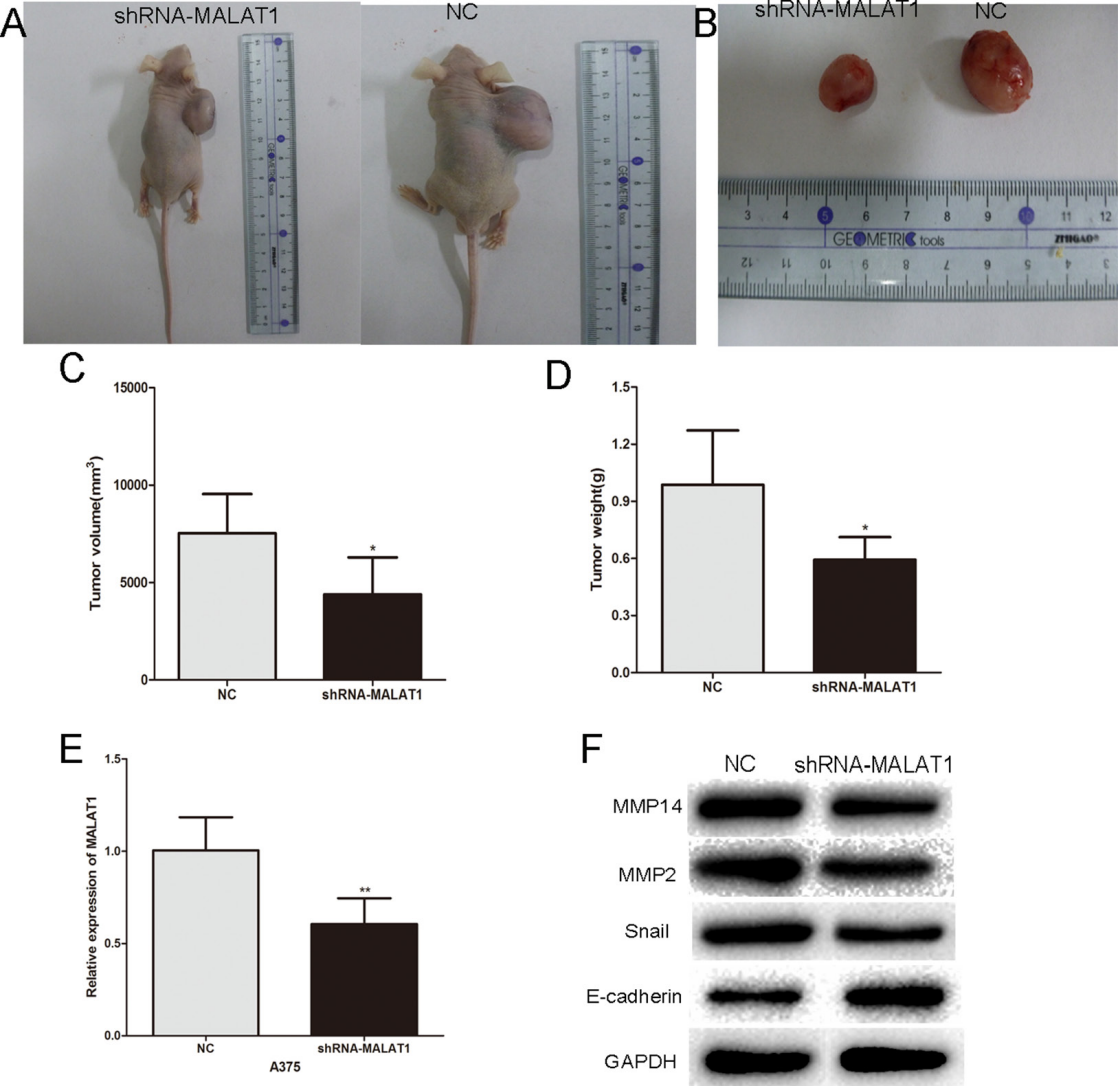
Inhibition of endogenous MALAT1 expression by siRNA inhibits melanoma growth *in vivo* **A.** and **B.** Tumor formation in nude mice and the excision tumor of A375 xenografts. **C.** The tumor volume of excision tumor by the formula: V(mm3) =0.5 * a* b^2^ (a represents the longest axis and b the shortest axis). **D.** The tumor weight of excision tumor. **E.** PCR identified MALAT1 expression changes. **F.** Western blots identified MMP14, Snail, E-cadherin and MMP2 protein expression changes. *P < 0.05, **P < 0.01, ***P<0.001.

To test the function of MALAT1 *in vivo*, nude mice were subcutaneously inoculated A375 cells stably expressing control shRNA or shRNA-MALAT1, respectively. After 4 weeks, the mice were sacrificed. The tumors were stripped and weighed. The average tumor weight in the control shRNA group was significantly higher than that in the shRNA-MALAT1 treated group. Meanwhile, the average tumor volume in the control group was significantly bigger compared with the shRNA-MALAT1 group (Figure 6A–6D). Finally, western blotting revealed that the expression of MMP14, MMP2 and Snail was reduced in shRNA-MALAT1 treated group, while E-cadherin protein expression was increased (Figure 6E and 6F). These results indicated MALAT1 may promotes melanoma progression *in vivo* by operating as a ceRNA.

## DISCUSSION

LncRNAs, ranging from 200 nucleotides to over 10 kb, have no significant role in coding protein. Studies of lncRNAs have achieved significant progress in the last decade. Many studies have shown that the aberrant expression of lncRNAs may play a critical role in the development of different tumors [26]. However, the functions and mechanisms of many lncRNAs are still unknown. Recently, many reports have shown that some specific endogenous lncRNAs can act as competing endogenous RNAs (ceRNA), which interfere with the miRNA pathways and influence post-transcriptional regulation [18, 19, 27]. The ceRNAs are implicated in the biological process of cancer, such that the disequilibration of ceRNAs and miRNAs can be critical for tumorigenesis [19, 27]. The ceRNAs is implicated in the biological process of cancer, the disequilibration of ceRNAs and miRNAs can be critical for tumorigenesis [27, 28]. For example, lncRNA-ATB was over-expressed in hepatocellular carcinoma and up-regulated ZEB1 and ZEB2 by competitively binding the miR-200, thus inducing invasion [29]. Similarly, lncRNA-H19 also acted as a ceRNA to modulate the availability of let-7. [30] Additionally, lncRNA-BC032469 can up-regulate hTERT expression by sponging miR-1207-5p and promotes proliferation in gastric cancer [31]. Thus, it is important to further understand the intricate networks of ceRNAs, leading to significant insight into gene regulatory networks in tumorigenesis.

MALAT1, a prognostic marker for lung cancer metastasis, is an lncRNA that was first found in non-small cell lung cancer [13]. Many studies have shown that MALAT1 is over-expressed in many solid tumors and associated with the proliferation and metastasis of several other cancers [14, 15, 32]. Accumulating evidences indicates that MALAT1 also promotes cancer progression by acting as a ceRNA. MALAT1 can induce breast cancer cell migration and invasion by sponging miR-1 [20]. MALAT1 also modulates radiosensitivity of HR-HPV+ cervical cancer though competitively binding miR-145 [22]. Some researchers have discovered that MALAT1 is highly expressed in melanoma and may have a correlation with melanoma metastasis [16]. However, the function and molecular mechanism of MALAT1 in melanomagenesis remains undefined. In this study, we report that MALAT1 is up-regulated in melanoma and promotes melanoma cell proliferation, invasion and migration. The potential miR-22 binding sites in MALAT1 transcripts were predicted. We subsequently demonstrated that MALAT1 is negatively regulated by miR-22 and that MALAT1 could act as a ceRNA by sponging miR-22 in melanoma cells.

MiR-22 is a tumour suppressor in many human tumors, such an renal cell carcinoma, glioblastoma, and gastric cancer [17, 33, 34]. However, little is known about the role of miR-22 in melanoma. Here, we found that miR-22 was largely reduced in melanoma tissues and acts as a tumor suppressor in melanoma cells. MMP14 and Snail are the direct functional effectors of miR-22. MMP14 can cleave a variety of substrates, including cell surface proteins (such as CD44) and other MMPs (such as pro-MMP2 and pro-MMP13), and induce ECM degradation and tumor cell invasion by increasing the secretion of other MMPs [24, 35]. Some research confirmed that Snail facilitates tumor invasion and metastasis by inhibiting E-cadherin and inducing EMT [25]. And lastly, we found that MALAT1 promoted melanoma cell growth and metastasis by competitively binding the miR-22, up-regulating MMP14 and Snail, and having downstream effects on MMP2 and E-cadherin protein. MALAT1 may promotes melanoma progression *in vivo* by operating as a ceRNA.

In conclusion, we demonstrated that MALAT1 is an oncogene in melanoma. MALAT1 can promote the growth and metastasis of melanoma cells though sponging miR-22, functionally releasing MMP14 and Snail mRNA transcripts targeted by miR-22, causing MMP2 activation and E-cadherin down-regulation, and inducing ECM remodelling and EMT. Understanding the regulatory mechanism of MALAT1 in melanoma could lead to the identification of novel therapeutic targets for melanoma. Future studies to assess the role of the MALAT1/ miR-22/ MMP14/ Snail axis in a clinical context are warranted.

## MATERIALS AND METHODS

### Tissue samples

Twenty primary malignant melanoma tissues and adjacent normal tissues (ANTs) were obtained from patients with malignant melanoma. They underwent surgical resection at The Affiliated People's Hospital of Jiangsu University, and the clinical pathological features of the patients were independently diagnosed by two professional pathologists. Every sample were frozen and stored in liquid nitrogen immediately after collection. The study was approved by the Ethics Committee of the Affiliated People's Hospital of Jiangsu University.

### Cell lines and cell culture

The human malignant melanoma cell lines A375 were purchased from the Chinese Academy of Sciences Cell Bank (Shanghai, China) and were maintained in Dulbecco's modified Eagle's medium (Gibco, USA) supplemented with 10% foetal bovine serum (Invitrogen, USA) and antibiotics (100 U/ml penicillin and 100 μg/ml streptomycin). The human malignant melanoma cell lines SK-MEL-5 and SK-MEL-2 were provided by American Type Culture Collection (ATCC, USA) and maintained in RPMI medium (Gibco, USA) supplemented with 10% FBS and penicillin/streptomycin. The human epidermal melanocytes HEMa-LP were obtained from Invitrogen (USA) and grown in medium 254 and HMGS (Cascade Biologics). The cell lines were incubated in an atmosphere containing 5% CO2 at 37°C.

### Plasmid constructs, Oligonucleotides and cell transfection

Oligonucleotides were purchased from GenePharma (Shanghai, China). The sequences were as follows:MALAT1-small interfering RNA-1 (si-MALAT1-1), 5′-GGCAAUGUUUUACACUAUUTT-3′; MALAT1-small interfering RNA-2 (si-MALAT1-2), 5′- CACAGGGAAAGCGAGTGGTTGGTAA-3′; negative control (NC), 5′-UUCUCCGAACGUGUCACGUTT-3′. The hsa-miR-22 mimic and hsa-miR-22 inhibitor were also purchased from GenePharma. The following short hairpin RNA (shRNA) was used to target human MALAT1: sense: 5′-CACAGGGAAAGCGAGTGGTTGGTAA-3′. The sequence of the negative control shRNA was 5′-TTCTCCGAACGTGTCACGT-3′. The shRNAs were synthesized and inserted into the pHBLV-U6 lentivirus core vector (Hanbio, Shanghai, China). For constructing MALAT1, MMP14 and Snail vectors, the full length of MALAT1 was amplified and inserted into pcDNA3.1 vectors (Invitrogen, USA), the full open reading frame cDNA clones for MMP14 and Snail were transcribed, and the products were amplified. The DNAs were inserted into pcDNA3.1. Oligonucleotides and constructs were transfected into melanoma cell lines by using Lipofectamine 2000 (Invitrogen, USA) according to the manufacturer's instructions.

### RNA isolation and quantitative real-time PCR

RNA was isolated from cells and tissues using TRIzol (Invitrogen, USA), following the manufacturer's instructions. Quantitative RT-PCR was used to detect the levels of MALAT1, MMP14, Snail, miR-18b, miR-1, miR-145 and miR-22. Reverse transcription (RT) was conducted with the Fermentas reverse transcription reagents and the Applied Biosystems® TaqMan® MicroRNA Reverse Transcription Kit (Applied Biosystems, CA). The ABI StepOnePlus system (Applied Biosystems, CA) was used to perform the amplification reaction according to predetermined conditions. The miR-18b, miR-1, miR-145 and miR-22 specific primer was purchased from Guangzhou Ribo BioCoLTD (Guangzhou, China) and U6 was used for normalization. To assess the levels of MALAT1, MMP14 and Snail, GAPDH was used for normalization. The 2^−ΔΔCt^ method was applied to analyse the data, and each experiment was performed in triplicate.

### Cell counting kit-8 (CCK-8) assay

The CCK-8 (Beyotime, China) assay was used to assess the proliferative ability of melanoma cells. The transfected A375 cells (5×10^3^ cells) were seeded into 96-well plates in 100 μl of culture media. The medium of each well was subsequently replaced with 100 μl of fresh culture media with 10 % CCK8 at different times (12, 24, 36 and 48 h) and then the cells were incubated for an additional 3 h. The absorbance was measured at an optical density of 450 nm using a microplate reader (Multiscan FC, Thermo Scientific). The experiments were independently repeated in triplicate.

### *In vitro* cell invasion and migration assays

Melanoma cell invasion was determined using the transwell assay. Transfected A375 cells were placed on the top of the Matrigel-coated invasion chambers (BD Biosciences, USA) with serum-free DMEM. In the lower chamber, we added 500 μl of DMEM containing 10% foetal bovine serum as a chemoattractant. After 24 h, cotton swabs were used to remove the non invasive cells. The invading cells were fixed with 95% ethanol, stained with 0.1% crystal violet, and counted and photographed under an inverted microscope (×100). The scratch wound assay was used to assess cell migration ability. First, transfected A375 cells were seeded into 6-well plates. After 24 h, cell layers were scratched using a 200 μl pipette tip to form wound gaps, and then the cells were maintained in 10% FBS-supplemented DMEM. The cells were photographed (0 and 24 h) under an inverted microscope to record the wound width. The experiments were independently repeated in triplicate.

### Luciferase reporter assay

We predicted the binding sites of miR-22 on MALAT1, MMP14 and Snail by using bioinformatics websites. The 3′-UTR fragment of MMP14 and Snail containing the putative miR-22 binding sequences was inserted into pMIR-REPORT vectors. The fragment (5383bp to 5431bp) of MALAT1 including the binding site (5403bp to5421bp) was inserted into pMIR-REPORT vectors. The mutated plasmid was used as the mutated control. The cells were co-transfected with the luciferase reporter construct and hsa-miR-22 mimic or mimic control. At 48 h following transfection, luciferase activity was detected using the Dual Luciferase Reporter Assay System (Promega, USA) according to the manufacturer's instructions.

### Western blot analysis

Total proteins were extracted from tissues and cells using RIPA buffer (KenGEN, China). Protein concentrations were quantified with a BCA Protein Assay Kit (Beyotime, China). Protein were separated by 10% SDS-PAGE and transferred to PVDF membranes (Millipore, USA). The membranes were blocked with 5% skim milk for 1 h. Subsequently, membranes were incubated overnight at 4°C with diluted antibodies against MMP14 (1:1000, Abcam, UK), Snail (1:1000, Abcam, UK), MMP2 (1:1000, Abcam, UK), or E-cadherin (1:1000, Abcam, UK), followed by incubation with an HRP-conjugated secondary antibody (1:2500, Santa Cruz, USA). GAPDH was used as a control (1:1000, CST, USA).

### RNA pull-down by MS2-MBP

Maltose-binding protein (MBP)-affinity purification was used to identify miRNAs that associated with MALAT1. The MS2-MBP protein was expressed and purified from E. coli following the protocol of the Steitz laboratory. Three bacteriophage MS2 coat protein-binding sites (5′- cgtacaccatcagggtacgagctagcccatggc gtacaccatcagggtacgactagtagatctcgtacaccatcagggtacg-3′) were inserted downstream of MALAT1 by site-directed mutagenesis using the Stratagene Quik Change Site Directed Mutagenesis Kit. To obtain miRNAs associated with the MS2-tagged MALAT1, the malignant melanoma cell line A375 was transfected with MS2-tagged MALAT1 constructs. Ten million cells were used for each immunoprecipitation assay. At 48 h following transfection, the cells were subjected to RNA pull-down analysis as described elsewhere [36].

### Xenograft tumor assay

Ten immunodeficient female nude mice (Beijing Laboratory Animal Center, Beijing, China) were used to test the effects of MALAT1 in malignant melanoma *in vivo*. This nude mice were randomly divided into two groups (5 mice/group). About 2×10^6^ logarithmically growing A375 cells stably expressing control shRNA or shRNA-MALAT1 were subcutaneously injected in nude mice, respectively. Subcutaneously. After 4 weeks, the tumors were stripped and weighed. Tumor volume was calculated according to the formula: V(mm^3^) =0.5 * a* b^2^ (a represents the longest axis and b the shortest axis). Total proteins and RNA were extracted from tissues, the expression of MALAT1, MMP14, Snail, MMP2 and E-cadherin were detected using western blot analysis or qRT-PCR.

### Statistical analysis

All experiments were independently repeated in triplicate. The data are presented as the mean ± standard error and were analysed with SPSS 10.0. Statistical evaluation of the data was performed by t-test (two-sided) and one-way-ANOVA. A P-value of less than 0.05 was considered statistically significant.
